# P‐wave dispersion is a vectorial phenomenon: Is it time to change minds?

**DOI:** 10.1002/joa3.12779

**Published:** 2022-09-13

**Authors:** Raimundo Carmona Puerta, Elizabeth Lorenzo Martínez, Ionut Donoiu, Elibet Chávez González

**Affiliations:** ^1^ Department of Electrophysiology and Arrhythmology Cardiovascular Hospital "Ernesto Guevara" Santa Clara Cuba; ^2^ Department of Physiology Medical University of Villa Clara Santa Clara Cuba; ^3^ Department of Cardiology University of Medicine and Pharmacy of Craiova Craiova Romania

**Keywords:** local theory, P wave dispersion, risk factor, vectorial theory

P‐wave dispersion (PWD) is an electrocardiographic parameter for predicting atrial fibrillation.^1^ There are two theories to explain the origin of PWD, one of them is the most acclaimed by researchers (local or conduction theory). Local theory proposes the presence of different conduction velocity zones in atrial wall leading to different P‐wave durations in the 12‐lead ECG. The other is vectorial theory, which argues, that any vector generated during the atrial depolarization process is projected unequally in the axes corresponding to the ECG leads. The conduction time of the P wave varies from the 12‐leads, and the records of 12‐lead ECG depend on the rotation of the heart in each patient. With vectorial theory, the maximum P wave (Pmax) and minimum P‐wave (Pmin) can be identified too. With the vectorial theory, both the maximum (Pmax) and minimum (Pmin) can be also identified.

Preferences with the local theory lie in the explanation of the relationship between PWD and atrial fibrillation (AF), the asynchrony of atrial wall depolarization is explained for this association.^1^, ^2^ The mechanism of PWD has remained unclear for more than 20 years. Recently it has been elucidated. New findings radically change the way to understand PWD in the clinical setting^.^ New knowledge leads us to wonder if the PWD parameter is still useful.^3^

Recently, local and vector theories were investigated at the same time in the same sample for analyzing the occurrence of the PWD phenomenon.^3^ This research could help us understand that the lead‐containing the Pmax is parallel to the electrical axis of the P wave, as well as, the lead‐expressing the Pmin is perpendicular to the P wave axis. The expression above justifies the inter‐lead differences between the P wave measured on the 12‐lead surface electrocardiogram and explains the different degrees of vector projection on the leads axes. In addition, it was found that PWD tends to dissipate when P wave is magnifyed(magnified) from 10× to 160×. After magnification, PWD was reduced from 48 to 4 ms.^3^

A moderate or high correlations between atrial conduction times and PWD has been demonstrated by echocardiography.^4^ However, at least three aspects allow us to think that it cannot be taken as evidence of a significant role of local theory to explain PWD. First, there have been no studies designed in the international literature to explore the value of the local theory, so the information concerning this issue is a secondary outcome; second, invasive electrophysiological measurements were not used; nevertheless, invasive electrophysiological study is universally accepted as the gold standard for quantifying cardiac conduction time and; third, none of these echocardiography studies determined the heterogeneity of atrial depolarization, which is the electrical phenomenon that theoretically gives rise to PWD, according to local theory.

All evidence points to vector theory as the main mechanism for producing PWD, On the other hand, conduction theory can explain the phenomenon but only weakly.^3^, ^5^ PWD appears as a consequence of the standardization of the 12‐lead electrocardiogram (25 mm/s ‐ 10 mm/mV or 50 mm/s ‐ 20 mm/mV) and disappear when using magnification software.^3^

Why does PWD continue to be a predictor parameter in clinical studies? A strong correlation between Pmax and PWD has been well described and it was later ratified.^1^, ^3^ Pmax measured on ECG is the variable most closely to the real value of total conduction time in the atrial wall and it is the best noninvasive assessor of atrial conduction time.^3^

That is why, the future of AF predictors ECG‐derived will rest on the so‐called interatrial blocks, which are the best possible expressions of the atrial conduction disturbances. Vectorial theory is the best explanation for the origin of PWD. Inhomogeneous atrial conduction the basis of local theory has a small but significant contribution to the origin of PWD, but only when there is delayed interatrial conduction. The vectorial justification of the PWD advise against it as a useful parameter for clinical use.^3^, ^5^

The authors who wrote this letter have been working for several years on the local theory. Vector theory had not been studied in the same sample of patients. Finally, we studied which of the two theories was the most essential. The role of the local and vectorial theories was demonstrated.^3^, ^5^ PWD phenomenon is only explained by vector theory when there is normal atrial conduction. And both the local and vector theories explain the phenomenon when there are conduction disturbances, but the vector theory explains it with more strength.^3^, ^5^

Certainly, there are limitations to PWD with the limited spatial resolution and the specified 12‐lead ECG, but there may be a future for PWD with improved spatial resolution and using other than usual 12‐lead ECG, such as mechanical analysis or rather than 12‐lead ECG. A change of mind is necessary and there is scientific evidence for it.

## CONFLICT OF INTEREST

The authors have no conflict of interest to declare.
